# Size of HIV‐1 reservoir is associated with telomere shortening and immunosenescence in early‐treated European children with perinatally acquired HIV‐1

**DOI:** 10.1002/jia2.25847

**Published:** 2021-11-19

**Authors:** Annalisa Dalzini, Giovanni Ballin, Sara Dominguez‐Rodriguez, Pablo Rojo, Maria Raffaella Petrara, Caroline Foster, Nicola Cotugno, Alessandra Ruggiero, Eleni Nastouli, Nigel Klein, Stefano Rinaldi, Savita Pahwa, Paolo Rossi, Carlo Giaquinto, Paolo Palma, Anita De Rossi, EPIICAL Consortium

**Affiliations:** ^1^ Section of Oncology and Immunology, Department of Surgery, Oncology and Gastroenterology University of Padova Padova Italy; ^2^ Hospital Universitario 12 de Octubre Madrid Spain; ^3^ Imperial College Healthcare NHS Trust London UK; ^4^ Research Unit of Clinical Immunology and Vaccinology Bambino Gesù Children's Hospital, IRCCS Rome Italy; ^5^ Department of Systems Medicine University of Rome Tor Vergata Rome Italy; ^6^ UCL Great Ormond Street Institute of Child Health London UK; ^7^ Department of Microbiology and Immunology, Miller School of Medicine University of Miami Miami Florida USA; ^8^ Department of Mother and Child Health University of Padova Padova Italy; ^9^ Immunology and Molecular Oncology Unit Veneto Institute of Oncology IOV – IRCCS Padova Italy

**Keywords:** HIV, paediatric HIV, immune ageing, telomeres, telomere length, early treatment

## Abstract

**Introduction:**

Persistence of HIV‐1, causing chronic immune activation, is a key determinant of premature senescence. Early antiretroviral therapy (ART) has been associated with a reduced HIV‐1 reservoir in children with perinatally acquired HIV‐1 (PHIV), but its impact on the senescence process is an open question. We investigated the association between HIV‐1 reservoir and biological and immune ageing profile in PHIV enrolled in the multicentre cross‐sectional study CARMA (Child and Adolescent Reservoir Measurements on early suppressive ART) conducted within the EPIICAL (Early treated Perinatally HIV Infected individuals: Improving Children's Actual Life) consortium.

**Methods:**

Between September 2017 and June 2018, CARMA enrolled 40 PHIV who started ART before 2 years of age and had undetectable viremia for at least 5 years before sampling date. Samples from 37 children with a median age of 13.8 years were available for this study. HIV‐1 DNA copies on CD4 cells, relative telomere length (marker of cellular senescence) and levels of T‐cell receptor rearrangement excision circle (TREC, marker of thymic output) on CD4 and CD8 cells were quantified by qPCR. Immunological profile was assessed by flow cytometry. Associations between molecular and phenotypic markers, HIV‐1 reservoir and age at ART initiation were explored using a multivariable Poisson regression.

**Results:**

Higher HIV‐1 reservoir was associated (*p*<0.001) with telomere shortening (incidence rate ratio [IRR] = 0.15 [0.13–0.17]), immunosenescence (CD28^–^CD57^+^, IRR = 1.23 [1.21–1.26]) and immunoactivation (CD38^+^ HLADR^+^, IRR = 7.29 [6.58–8.09]) of CD4 cells. Late ART initiation (after 6 months of age) correlated with higher HIV‐1 reservoir levels (552 [303–1001] vs. 89 [56–365] copies/10^6^ CD4 cells, *p* = 0.003) and percentage of CD4 senescent cells (2.89 [1.95–6.31] vs. 1.02 [0.45–2.69, *p* = 0.047). TREC levels in CD8 cells were inversely associated with HIV‐1 reservoir (IRR = 0.77 [0.76–0.79]) and were significantly lower in late treated PHIV (1128 [486–1671] vs. 2278 [1425–3314], *p* = 0.042).

**Conclusions:**

Later ART initiation is associated with higher HIV‐1 reservoir size, which correlates with increased telomere shortening and senescence of CD4 cells. Timing of ART initiation in infancy has long‐term consequences on the immune and biological ageing profile of children with perinatally acquired HIV‐1.

## 1 INTRODUCTION

Antiretroviral therapy (ART) has dramatically changed the natural history of patients living with HIV‐1 in terms of survival and quality of life [1]; however, it is unable to eradicate the virus, mainly because of the persistence of latently infected cells. Despite improved immune functions and reduced AIDS‐related complications, HIV‐1 positive patients on ART have a higher risk of non‐AIDS‐related morbidity and mortality compared to age‐matched HIV‐1 negative individuals, due to their increased incidence of a wide range of illnesses associated with premature ageing [2, 3, 4, 5].

Chronic immune activation by HIV‐1 is a key determinant of premature senescence: viral persistence induces the activation and continuous expansion of immune cells, that eventually reach the senescent stage and lose their functions [6], and leads to telomere shortening to a critical length under which the replicative capacity is lost [7]. The link between telomere shortening, cellular senescence and ageing is well established [8, 9]; shorter telomeres and telomere attrition are linked with increased risk and severity of cardiovascular diseases, stroke, heart attack and mortality [5]. HIV‐1 itself can impair the activity of telomerase, in particular in infected CD4 cells [10, 11], increasing the apoptotic propensity of haematologic cells, leading to immune system dysfunction. Accelerated shortening of telomeres may also be an adverse effect of nucleoside reverse transcriptase inhibitors in ART [12, 13]. However, studies indicate that HIV‐1 *per se*, rather than exposure to ART, is responsible for accelerated telomere shortening [14, 15].

To date, few data are available on premature ageing in children with perinatally acquired HIV‐1 (PHIV) [3, 5, 14, 16]. Exposure to the virus from birth may accelerate premature ageing and immunosenescence in HIV‐1 positive infants. Cross‐sectional studies indicated that telomere length in peripheral blood cells is shorter in HIV‐1 positive children and adolescents compared to age‐matched HIV‐1 negative subjects [14, 17, 18], and within the HIV‐1 positive children, telomeres are shorter in viremic children compared to aviremic ones [14, 19]. Moreover, HIV‐1 positive children display higher percentages of activated and senescent CD8 cells, which inversely correlate with telomere length [14], thus supporting the link between biological ageing and immunosenescence.

Initiation of ART soon after infection preserves adaptive immune functions [20, 21] with a decrease of residual viral replication and consequent reduction of viral reservoir size [22], but its impact on the senescence process is still an open question. This study is part of the CARMA (Child and Adolescent Reservoir Measurements on early suppressive ART) study, which aims to identify biomarkers associated with viral reservoir size in a unique cohort of European PHIV on long‐term suppressive ART [23, 24, 25], who may represent optimal candidates for future studies on immunotherapeutic strategies for prolonging ART‐free viral remission [1]. In this context, our aim was to investigate the association between HIV‐1 reservoir and biological and immune ageing profile in PHIV enrolled in the CARMA study.

## 2 METHODS

### 2.1 Study population

Forty PHIV who started ART within 2 years of life were enrolled, between September 2017 and June 2018, within the cross‐sectional study CARMA [23, 24, 25]. Enrolment criteria required viral suppression (<400 copies HIV RNA/ml) achieved within 12 months of ART initiation, maintained for a minimum of 5 years (<50 copies HIV RNA/ml) and confirmed at enrolment. Viral load was recorded at diagnosis, ART initiation, throughout treatment and at time of sampling. Blips (defined as a rise in plasma viremia from 50 to 399 copies/ml returning to <50 copies/ml on repeated sampling) and a single annual spike (defined as 400–999 copies/ml returning to <50 copies/ml on repeated sampling) were permitted. For the present study, samples from 37 out of 40 enrolled PHIV were available.

The CARMA study, conducted within the EPIICAL Consortium (Early treated Perinatally HIV Infected individuals: Improving Children's Actual Life; www.epiical.org) [1], is a multicentre global collaboration that involves seven European paediatric clinical research centres; it was approved by the local institutional ethic committees [23] of Bambino Gesù Children's Hospital (Rome, Italy), University of Padova (Padova, Italy), University Hospital 12 de Octubre and Hospital Gregorio Marañón (Madrid, Spain), St Mary's University (Twickenham, UK), Great Ormond Street Hospital (London, UK), Brighton and Sussex University Hospitals (Brighton, UK). Study participants or their legal guardians gave written informed consent in accordance with the Declaration of Helsinki.

### 2.2 Sample preparation

Blood samples were collected in EDTA‐containing tubes and processed within 12 hours from the blood draw. Peripheral blood mononuclear cells (PBMCs) were isolated by centrifugation on a Ficoll‐Paque gradient (Pharmacia, Uppsala, Sweden) and stored in liquid nitrogen until use.

### 2.3 Isolation of CD4 and CD8 T‐cell subsets

CD4 and CD8 cell subsets were isolated from PBMC with CD4^+^ T Cell Isolation Kit, human and CD8^+^ T Cell Isolation Kit, human (Miltenyi Biotec, Auburn, CA, USA) following manufacturer's instructions and stored at –20°C until use. The purity of the enriched fraction was checked for a selection of samples with flow cytometry by staining with fluorescent‐conjugated mononuclear antibodies CD3‐fluorescein isothiocyanate (FITC), CD4‐peridinin chlorophyll protein (PerCP) and CD8‐Viogreen.

### 2.4 DNA extraction

DNA was extracted from PBMC and the purified CD4 and CD8 cell subsets using QIAamp DNA Mini Kit (Qiagen, Hilden, Germany) following manufacturer's instructions. DNA concentration was estimated with Implen Nanophotometer 15920.

### 2.5 HIV‐1 DNA quantification on CD4 T cells by droplet digital PCR

HIV‐1 DNA levels in purified CD4 T cells were measured by the QX200™ Droplet Digital™ PCR (ddPCR) system (Bio‐Rad, Pleasanton, CA, USA). The ddPCR mix was prepared mixing extracted DNA with 2X ddPCR Supermix for Probes (Bio‐Rad); LTRfw and LTRrv primers [26], or hTERTfw and hTERTrv primers [27], and LTR or hTERT probe, respectively. Droplets were formed in the QX200™ Droplet Generator (Bio‐Rad), then placed into a 2720 Thermal Cycler (Applied Biosystem) with the following cycling conditions: 94°C for 10 minutes; 45 cycles at 94°C for 30 seconds and 58.5°C for 1 minute; and 98°C for 10 minutes. The droplets were then read by the QX200™ Droplet Reader (Bio‐Rad) and the results were analysed with the QuantaSoft™ Analysis Software 1.7.4.0917 (Bio‐Rad). Wells with less than 10,000 droplets were discarded from the analysis. Each sample was run in triplicate. The HIV‐1 copy number was normalized to the hTERT copy number, and the results were expressed as HIV‐1 DNA copies/10^6^ CD4 cells.

### 2.6 Telomere length measurement by quantitative real‐time PCR

Relative telomere length (RTL) was determined on DNA extracted from PBMC, purified CD4 and purified CD8 cells by multiplex quantitative real‐time PCR, as previously described [14, 28]. All DNA samples and reference samples were run in triplicate. Results were analysed using LinRegPCR free software [29]. Telomere length values were calculated as telomere/single‐copy gene ratio [14, 28].

### 2.7 Quantification of T‐cell receptor rearrangement excision circle levels

Thymic output in PBMC, in purified CD4 and CD8 cell subsets, was evaluated by the measurement of T‐cell receptor rearrangement excision circle (TREC) levels by real‐time PCR, as previously described [30]. TREC levels were expressed as the number of TREC copies/10^5^ copies of PBMC, CD4 or CD8 T cells.

### 2.8 Flow cytometry analysis

T‐cell phenotyping was performed on cryopreserved PBMC. Cells were thawed, washed and stained in the dark with fluorescent‐conjugated mononuclear antibodies CD3‐FITC, CD4‐peridinin chlorophyll protein (PerCP), Human Leukocyte Antigen D Related (HLA‐DR)‐allophycocyanin (APC), CD38‐phycoerythrin (PE), CD57‐PE (Becton‐Dickinson, San Diego, CA, USA), CD8‐Viogreen and CD28‐APC (Miltenyi Biotec). Samples were analysed using LSRII Flow cytometer (Becton‐Dickinson). A total of 100,000 events were collected in the lymphocyte gate using morphological parameters (forward and side‐scatter). Data were processed with FACSDiva Software (Becton‐Dickinson) and analysed using Kaluza Analysis Software v.1.2 (Beckman Coulter). Live/Dead Fixable Near‐IR Dead Cell Stain Kit (Life Technologies, Carlsbad, CA, USA) was employed to stain and exclude dead cells.

### 2.9 Statistical analysis

In the summary tables, continuous variables were described as medians and interquartile ranges (IQRs) and absolute numbers and relative frequencies when categorical.

Children were subgrouped according to HIV‐1 reservoir levels: children with viral DNA levels below the first interquartile (75 copies/10^6^ CD4 cells) were assigned to the “low HIV‐DNA” group, while children with viral DNA levels above the first interquartile were assigned to the “high HIV‐DNA” (see Table S1). Children were also subgrouped into “early‐treated” if they started ART before 6 months of age and “late‐treated” if they started ART between 6 and 24 months of age. Comparisons between low and high HIV‐1 DNA subgroups and between early‐ and late‐treated groups were assessed by the Wilcoxon test and Fisher's exact test when appropriate. Correlations were explored using Spearman's ρ test.

To assess the association between HIV‐1 DNA reservoir and immunological and biological parameters, we performed a multivariable Poisson regression adjusted by pre‐ART viral load, pre‐ART % of CD4 and age at reservoir measurement. The age at ART was included in the model as an interaction term. Variable selection for the models was done by a backwards stepwise procedure using Akaike index criterion. Variables, such as age at HIV‐1 diagnosis, ART regimen at interaction, ethnicity and gender, were not included in the best‐fitted model. These associations are interpreted *via* the incidence rate ratio (IRR): an IRR above 1 determines a positive association between HIV‐1 reservoir and the predictor, while an IRR below 1 determines an inverse association between them.

All the analyses were conducted using R Software (R Core Team [2014]. R: A language and environment for statistical computing. R Foundation for Statistical Computing, Vienna, Austria. URL http://www.R‐project.org/).

## 3 RESULTS

### 3.1 Maximizing the sensitivity of HIV‐1 reservoir quantification

The 37 children included in this study started ART at a median of 4.1 [IQR 2.5–6.2] months of age; the median time at sampling was 13.5 [7.8–16.4] years after ART initiation. Virological and immunological findings are shown in Table 1. Total HIV‐1 DNA content was 255 [75–434] copies/10^6^ CD4 cells. The employment of ddPCR on isolated CD4 cells to detect HIV‐1 reservoir increased the sensitivity compared to the measurement on PBMC; indeed, only five out of 37 samples exhibited HIV‐1 DNA levels <10 copies/10^6^ CD4 cells and values were, on average, five‐fold higher than those measured on total PBMC (255 [75–434] HIV‐1 DNA copies/10^6^ CD4, this work vs. 48 [7–112] HIV‐1 DNA copies/10^6^ PBMC [23]); the two sets of values were strongly correlated (ρ = 0.735, *p* < 0.0001).

**Table 1:** Clinical, immunological and virological characteristics of the studied cohort at sample collection

	Overall	*N*
Male/female	12/25	37
Age (years)	13.8 [9.0–16.7]	37
Time on ART (years)	13.5 [7.8–16.4]	37
% CD4	32.7 [28.0–39.1]	37
% CD8	15.3 [12.6–18.4]	37
HIV‐1 DNA copies/10^6^ CD4 cells	255 [75–434]	35
CD28^–^ CD57^+^ senescent CD4 cells, %	1.63 [0.58–2.90]	35
CD28^–^ CD57^+^ senescent CD8 cells, %	12.7 [8.0–16.8]	35
CD38^+^ HLADR^+^ activated CD4 cells, %	0.37 [0.27–0.54]	35
CD38^+^ HLADR^+^ activated CD8 cells, %	1.55 [1.00–2.01]	35
TREC levels/10^5^ PBMC	1720 [846–2730]	37
TREC levels/10^5^ CD4 cells	1314 [955–2084]	36
TREC levels/10^5^ CD8 cells	1911 [1082–3256]	37
Relative telomere length in PBMC	1.34 [1.20–1.44]	37
Relative telomere length in CD4 cells	1.33 [1.222–1.57]	36
Relative telomere length in CD8 cells	1.40 [1.24–1.52]	35

*Note*: Values are expressed as median [interquartile range]. Abbreviations: ART, antiretroviral therapy; PBMC, peripheral blood mononuclear cell; TREC, T‐cell receptor rearrangement excision circle.

### 3.2 Higher HIV‐1 reservoir correlates with older age at ART initiation and unfavourable ageing profile

**Figure 1:**
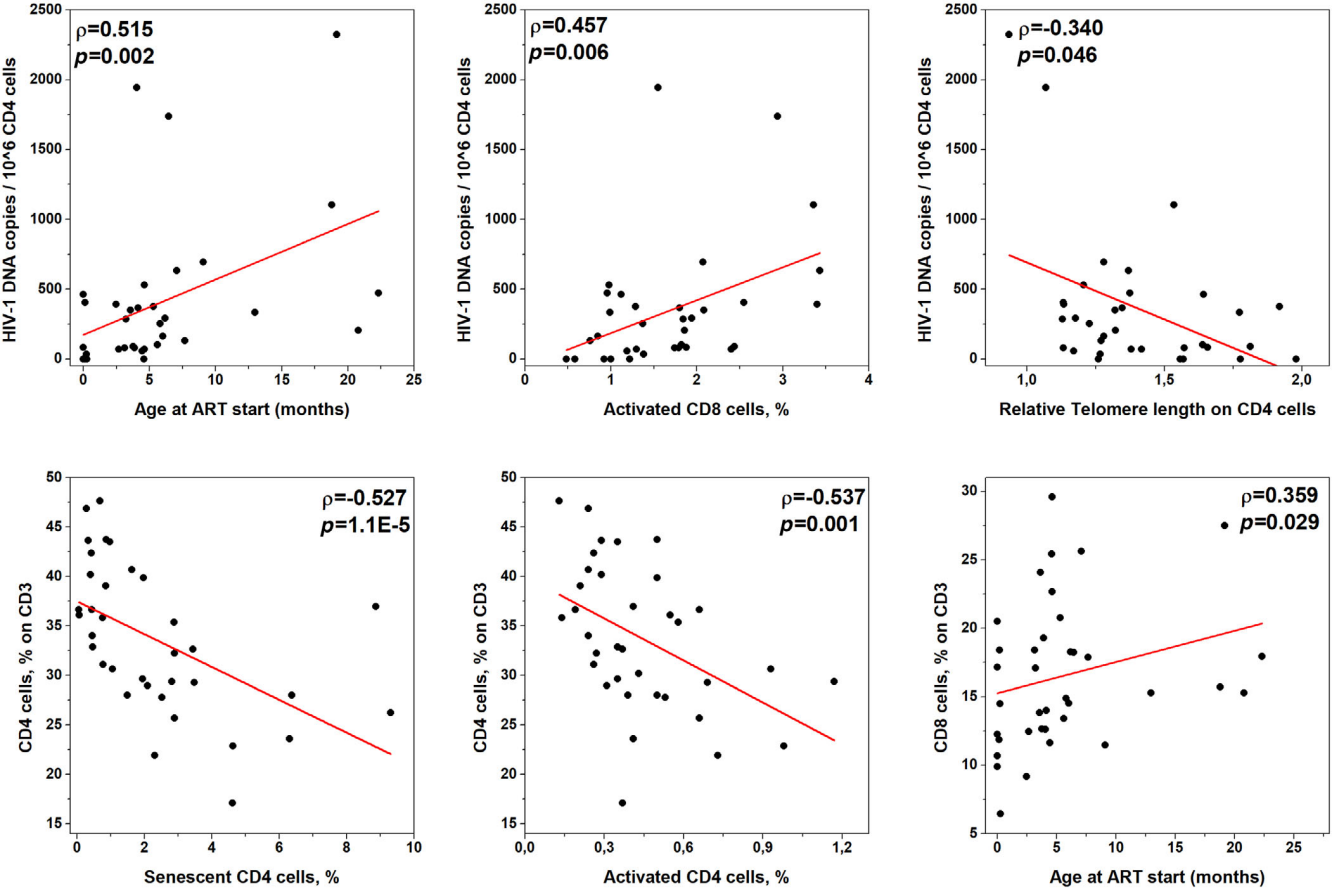
Correlations with HIV‐1 DNA. Correlations between HIV‐1 DNA copies (first row) or percentage of CD4 and CD8 cells (second row) at time of sampling, and relevant virological and immunological characteristics of the studied cohort. Linear fit is represented with a red line. Spearman's ρ coefficient and its *p* value are reported in each panel.

Univariate analysis (Figure 1 and Figure S1) revealed that HIV‐1 reservoir correlates with the age at ART initiation (ρ = 0.515, *p* = 0.002), thus indicating that the later ART is initiated, the larger is the size of the HIV‐1 reservoir. HIV‐1 reservoir also correlated positively with the percentage of activated CD8 cells (CD38^+^ HLADR^+^, ρ = 0.457, *p* = 0.006) and inversely with telomere length in CD4 cells (ρ = –0.340, *p* = 0.046).

Later age at ART initiation correlated with higher percentages of CD8 (ρ = 0.467, *p* = 0.012) cells and lower percentages of CD4 cells (ρ = −0.405, *p* = 0.026) at the time of ART start; after about a decade on suppressive ART, later age at ART initiation still correlated with higher percentages of CD8 cells (ρ = 0.359, *p* = 0.029) and, slightly, with lower percentages of CD4 cells (ρ = −0.173, *p* = 0.307). Moreover, lower percentages of CD4 cells at sample collection correlated with higher percentages of senescent (CD28^–^ CD57^+^, ρ = −0.527, *p* < 0.001) and activated (ρ = −0.537, *p* = 0.001) CD4 cells.

**Figure 2:**
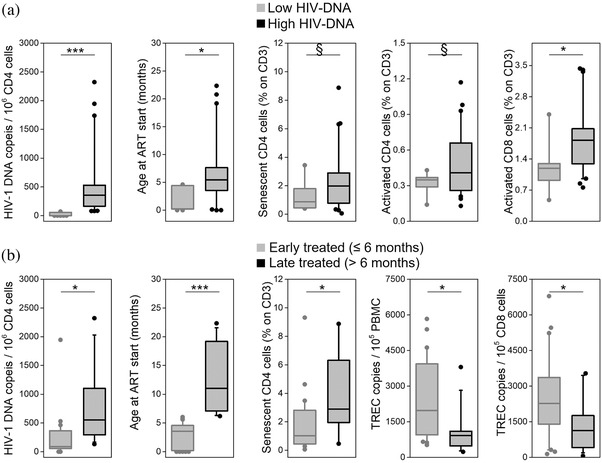
Comparison between relevant clinical, immunological and virological characteristics of the studied cohort subgrouped by (a) their HIV‐1 DNA levels or (b) their age at ART start. Early treated: ART initiation ≤6 months of age; late treated: ART initiation between 6 and 24 months of age. Boxes show median values and interquartile ranges; whiskers represent 5–95th percentile; outliers are plotted as dots. § *p* < 0.09; * *p* < 0.05; *** *p* < 0.001. Abbreviations: ART, antiretroviral therapy; PBMC, peripheral blood mononuclear cell; TREC, T‐cell receptor rearrangement excision circle.

To further understand how HIV‐1 reservoir shapes the immune profile, PHIV were subgrouped according to their HIV‐1 DNA levels into “low HIV‐DNA” and “high HIV‐DNA” (Figure 2a and Table S1). The two groups did not significantly differ for their age at sample collection and time under ART (Table S1).

PHIV in the low HIV‐DNA group started ART significantly earlier than the high HIV‐DNA group (1.46 [0.15–4.47] vs. 5.59 [3.65–8.37] months, *p* = 0.006), and they had higher percentage of CD4 cells at the beginning of treatment (37 [30–51] vs. 28 [18–38], *p* = 0.050). After over 10 years of suppressive ART, they displayed a significantly lower activation of CD8 cells (1.21 [0.84–1.48] vs. 1.83 [1.16–2.35], *p* = 0.030) and a tendency to have lower percentages of senescent (0.76 [0.42–1.80] vs. 2.15 [0.80–3.34], *p* = 0.060) and activated (0.33 [0.26–0.38] vs. 0.46 [0.26–0.66], *p* = 0.089) CD4 cells.

PHIV were also subgrouped according to their age at time of ART start (Figure 2b and Table S1): late‐treated children had significantly larger reservoir than early‐treated ones (552 [270–1262] vs. 89 [45–371] HIV‐1 DNA copies/10^6^ CD4 cells, *p* = 0.003), higher percentages of CD4 senescent cells (2.89 [1.32–6.34] vs. 1.02 [0.44–2.83], *p* = 0.048) and lower percentages of CD4 cells (27.9 [24.1–32.9] vs. 35.4 [29.8–40.0], *p* = 0.045). Moreover, they had lower TREC levels in PBMC and in CD8 cells (921 [498–1083] vs. 1976 [1075–3881] TREC/10^5^ PBMC, *p* = 0.008, and 1128 [480–1671] vs. 2278 [1425–3314] TREC/10^5^ CD8 cells, *p* = 0.040). The two groups did not significantly differ for their age and time under ART.

### 3.3 HIV‐1 reservoir is associated with senescence, activation and shortening of telomeres

Multivariate associations between HIV‐1 reservoir and markers of biological and immunological ageing were explored using a multivariable Poisson regression analysis adjusted by pre‐ART viral load, pre‐ART % CD4 and age at reservoir measurement (see Methods); age at ART initiation was included in the model as an interaction term.

**Figure 3:**
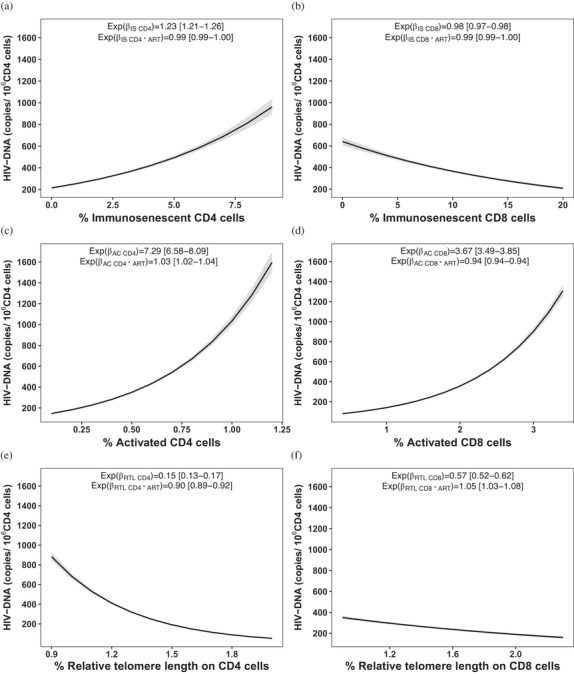
Associations with HIV‐1 DNA levels. Associations between HIV‐1 DNA levels in senescent CD4 cells (a), senescent CD8 cells (b), activated CD4 cells (c), activated CD8 cells (d), relative telomere length of CD4 cells (e) and relative telomere length of CD8 cells (f). Incidence rate ratio coefficients (Expβ) and their interaction with age at ART start (Expβ*age at ART start) were calculated using the multivariable Poisson regression model. The Expβ of each subset represents the association between this subset and the HIV‐1 reservoir adjusted by the age at ART initiation. The Expβ*age at ART start states the effect of this subset on HIV reservoir for every month elapsed without ART initiation. Predictors with a positive association with HIV‐1 DNA have an Expβ coefficient above 1; those with an inverse association have an Expβ coefficient below 1.

A strong positive association (Table 2 and Figure 3) was found between HIV‐1 reservoir and activated CD4 and CD8 cells, indicating that the higher is the immune activation, the higher is the HIV‐1 reservoir. In particular, a 1% increase in the percentage of activated CD4 cells is associated with a seven‐fold increase of the viral reservoir (IRR = 7.29 [6.58–8.09]) and a 1% increase in the percentage of activated CD8 cells is associated with an almost four‐fold increase of the viral reservoir (IRR = 3.67 [3.49–3.85]). These associations, respectively, increase by 3% (IRR _activated CD4*age at ART start_ =1.03 [1.02–1.04]) and decrease by 6% (IRR _activated CD8*age at ART start_ = 0.94 [0.94–0.94]), for each month the start of the therapy is delayed.

**Table 2:** Associations between HIV‐1 DNA levels in CD4 cells and tested HIV‐1 DNA predictors

Predictor	Incidence rate ratio (Expβ)	*p*	Effect of ART delay on incidence rate ratio (β_(cell*age at ART start)_)	*p*	*N*
Senescent CD4 cells, %	1.23 [1.21–1.26]	<0.001	0.99 [0.99–1.00]	<0.001	35
Senescent CD8 cells, %	0.98 [0.97–0.99]	<0.001	0.99 [0.99–1.00]	<0.001	35
Activated CD4 cells, %	7.29 [6.58–8.09]	<0.001	1.03 [1.02–1.04]	<0.001	35
Activated CD8 cells, %	3.67 [3.49–3.85]	<0.001	0.94 [0.94–0.94]	<0.001	35
TREC levels/10^5^ PBMC	0.74 [0.72–0.76]	<0.001	1.00 [0.99–1.00]	n.s.	37
TREC levels/10^5^ CD4 cells	1.05 [1.00–1.09]	n.s.	0.95 [0.94–0.95]	<0.001	36
TREC levels/10^5^ CD8 cells	0.77 [0.76–0.79]	<0.001	0.94 [0.94–0.94]	<0.001	37
Relative telomere length in PBMC	0.65 [0.59–0.71]	<0.001	0.90 [0.89–0.91]	<0.001	37
Relative telomere length in CD4 cells	0.15 [0.13–0.17]	<0.001	0.90 [0.89–0.92]	<0.001	36
Relative telomere length in CD8 cells	0.57 [0.52–0.62]	<0.001	1.05 [1.03–1.08]	<0.001	35

*Note*: Incidence rate ratio coefficients (Expβ) and their interaction with age at ART start (Cell*age at ART start) were calculated using the multivariable Poisson regression model. These regression models fit a log‐linear relationship. Predictors with a positive association with HIV‐1 DNA have an Expβ coefficient above 1; those with an inverse association have an Expβ coefficient below 1.

Abbreviations: ART, antiretroviral therapy; PBMC, peripheral blood mononuclear cell; TREC, T‐cell receptor rearrangement excision circle.

HIV‐1 reservoir was also strongly associated with senescent CD4 cells: for each 1% increase in the percentage of senescent CD4 cells, there is an increase of the viral reservoir of 23% (IRR = 1.23 [1.21–1.26]). In agreement, increasing RTL in CD4 cells by 1 unit is associated with a 85% reduction of the viral reservoir (IRR = 0.15 [0.13–0.17]); this association decreases by 10% for each month the start of therapy is delayed (IRR_RTL CD4*age at ART start_ = 0.90 [0.89–0.92]).

Only a mild inverse association was found between HIV‐1 DNA reservoir and senescent CD8 cells: a 1% increase in the percentage of senescent CD8 cells is associated with a decrease of 2% of the viral reservoir (IRR = 0.98 [0.97–0.99]). Moreover, increasing RTL in PBMC and CD8 cells by one unit is associated with a 35% and 43% decrease of viral reservoir, respectively (IRR = 0.65 [0.59–0.71] and IRR = 0.57 [0.52–0.62]). This effect decreases by 10% for each month the start of therapy is delayed (IRR_RTL PBMC*age at ART start_ = 0.90 [0.89–0.91]) in PBMC, while it increases by 5% (IRR_RTL CD8*age at ART start_ = 1.05 [1.03–1.08]) in CD8 cells.

An inverse association was found between TREC in PBMC and HIV‐1 reservoir: an increase in one unit of TREC (defined as 1000 copies/10^5^ PBMC) is associated with a decrease of 26% of the viral reservoir (IRR = 0.74 [0.72–0.76]); this association is not significantly influenced by a delay in ART initiation. Similarly, a one unit increase of TREC in CD8 cells is associated with a decrease of the viral reservoir of 23% (IRR = 0.77 [0.76–0.79]); this association decreases by 6% for each month the start of therapy is delayed (IRR _TREC CD8*age at ART start_ = 0.94 [0.94–0.94]).

## 4 DISCUSSION

The driving hypothesis of the CARMA study is that early initiation of ART in PHIV, combined with a continuous viral control with an effective therapy, could impact on the size of viral reservoir and be associated with long‐lasting biological and immunological favourable outcomes. Our results show that the earlier ART is initiated, the smaller is HIV‐1 reservoir size, and that a larger reservoir, associated with a later ART initiation, correlates with telomere shortening and senescence of CD4 cells.

It has been shown that adults [31] and children [22, 32] who started ART early after the primary infection have a reduced viral reservoir size: the detection of small reservoir poses a challenge for future strategies aiming at HIV‐1 remission that will most likely target individuals with long‐term viral suppression and reduced reservoirs. CD4 cells are the primary target of infection by HIV‐1 and resting ones are the best characterized latently infected cells that comprise the majority of the reservoir [33, 34]. In order to maximize our ability to detect HIV‐1 reservoir in our peculiar cohort, we measured HIV‐1 DNA on purified CD4 cells, which has greatly improved the sensitivity of HIV‐1 reservoir detection. Our results confirm the strong correlation between early treatment and reduced reservoir size [22, 23, 24, 25], independently from ART duration, indicating that a prompt ART initiation is fundamental for limiting the negative impact of the virus. Children with lower percentages of CD4 cells at the beginning of treatment display higher reservoir at sample collection; moreover, those who started ART later maintain higher reservoir levels, higher percentages of CD8 and lower percentages of CD4, even after a decade of treatment. This is in line with observations from a recent analysis of the long‐term consequences of planned treatment interruptions in PHIV, showing how even brief rebounds of viremia in usually well‐suppressed individuals have a negative impact at immunological level lasting for over a decade [35].

The persistence of HIV‐1 appears a key determinant of premature senescence [5]: despite long‐term ART, adults living with HIV have higher immune activation and senescence [4, 36] and are at greater risk of HIV‐associated non‐AIDS conditions, compared to the general population [37]. Studies in PHIV have shown a clear correlation between premature immune ageing, identified by the abnormal expansion of aged B‐ and T‐cell subsets, and a lower ability to mount and maintain specific immune responses [14, 16, 21, 38] and an impaired response to vaccinations [20, 39], highlighting the detrimental effect of precocious immune senescence in this population.

Studies reporting telomere shortening as a marker of immunosenescence over HIV‐1 infection present conflicting evidence. Shiau et al. [17] reported shorter absolute telomere length in HIV‐1 positive and HIV‐1 exposed negative children compared with HIV‐1 unexposed children. Conversely, Gianesin et al. [14] found that telomere length of HIV‐1 positive, HIV‐1 exposed negative and HIV‐1 unexposed children was significantly different. It appears reasonable that HIV‐1 infection could have a major detrimental impact on cellular ageing, while the negative effects of prophylaxis and ART are negligible compared to that of HIV‐1 *itself* [5]; however, the interplay between HIV‐1 and immune senescence, inflammation and immune activation is still an open question. A study in HIV‐1 positive adults indicates that HIV‐1 levels are associated with markers of inflammation and activation before ART, but not during long‐term ART, suggesting that HIV‐1 reservoir may be a consequence of pre‐ART immune activation and inflammation [40]. However, other studies show that even in the optimal situation of persistent viral suppression, a higher HIV‐1 reservoir is strongly associated with significantly more CD4 and CD8 activated cells [3, 5, 14], an observation confirmed by the present work.

Moreover, we found that both a higher HIV‐1 reservoir and a later treatment initiation from birth correlate with higher percentages of senescent CD4 cells; these observations are corroborated by the strong association between higher HIV‐1 reservoir and telomere erosion in PBMC, CD4 and CD8 cells. Interestingly, the strength of the association between HIV‐1 reservoir and telomere erosion in CD4 slowly declines for each month ART is delayed: this may be explained by the coexistence, over time, of other pro‐ageing factors that may impact on ageing in addition to HIV‐1. A recent study [2] reports a similar observation: HIV‐1 induced precocious B cells ageing was more prominent in younger compared to elderly HIV‐1 positive adults.

Thymic output is a key event in the immune reconstitution process occurring in children on ART and in the T‐cell homeostasis; TREC is a marker of thymic output and inversely correlates with age, thus TREC evaluation also represents a useful marker of immunosenescence [41, 42]. A study [43] on the role of thymic function on CD4 T‐cell maintenance, measured by the sj/β‐TREC ratio, was conducted in HIV‐1 adult progressors, long‐term non‐progressor and paediatric patients; results pointed out that age at infection is important to preserve thymic function, greatly supporting early ART initiation. In our study, delayed treatment correlates with lower TREC levels in PBMC and in CD8 cells; conversely, TREC levels in CD4 cells are not significantly affected, which could be an indication that, in children, CD4 turnover is stimulated due to the increased senescence of CD4 cells. It was described [44] that levels of cell‐associated HIV‐1 DNA are positively associated with TREC, suggesting the existence of a homeostasis between peripheral CD4 cells and thymic output, that is the higher the HIV‐DNA levels, the greater the peripheral CD4 depletion and, in turn, the greater the thymic output. A similar observation is reported in a study comparing young adults who acquired HIV‐1 perinatally or later in life [18]. This is also consistent with a work that has shown that there is a strong correlation between CD4 levels and both thymic output and naïve CD4 T cells [45].

This study is limited by sample availability: a single time point was available, which allowed only a cross‐sectional evaluation. Other limitations include the lack of evaluation of the size of intact provirus and the relatively small size of the cohort, which, however, reflects the limited portion of patients who achieve viral control in infancy and maintain it for decades. Nevertheless, our findings contribute to the field of paediatric HIV: to the best of our knowledge, telomere length, TREC levels, immune activation and immunosenescence have been investigated together for the first time in a cohort with the unique characteristics of the CARMA cohort. Our results demonstrate that timing of ART initiation is crucial, and its delay has long‐term consequences.

## 5 CONCLUSIONS

This study investigates for the first time the association between HIV‐1 reservoir and telomere shortening, thymic output and immunosenescence in a unique cohort of long‐term virally suppressed adolescents who initiated ART early in life. We show that early ART initiation restricts the size of viral reservoir and prevents premature immunosenescence and telomere shortening. Timing of ART initiation in infancy is crucial and has long‐term consequences on the immune and biological ageing profile.

## COMPETING INTERESTS

The authors declare no competing interests.

## AUTHORS’ CONTRIBUTIONS

AD and GB performed all the experiments. ADR designed the research study; AD and ADR designed the experimental plan. CG contributed essential reagents, tools and resources. AD analysed the data; SDR performed the statistical modelling and analysed the data. AD, ADR and MRP wrote the paper. AD, SDR, MRP, NC, AR, SR, SP, PP and ADR revised the paper. PR, CF, EN, NK, PR, CG, PP and ADR supervised the enrolment of the patients, provided clinical and virological data and contributed to scientific discussion.

## FUNDING

The CARMA was supported by the EPIICAL (Early‐Treated Perinatally HIV‐Infected Individuals: Improving Children's Actual Life With Novel Immunotherapeutic Strategies) project, funded through an independent grant by ViiV Healthcare United Kingdom. This work is part of the EPIICAL project (http://www.epiical.org/), supported by the PENTA‐ID foundation (http://penta‐id.org/). AD and MRP were supported by a fellowship from EPIICAL.

## Supporting information


**Figure S1**. Heatmap of the correlations between all the studied clinical, virological and immunological characteristics of the studied cohort. Correlations are colored according to their Spearman's ρ coefficient. § *p* < 0.1; *p < 0.05; **p < 0.01; ***p < 0.001.
**Table S1**. Comparisons between all the studied clinical, virological and immunological characteristics of the studied population, subgrouped according to the patients' HIV‐1 DNA values or by their age at ART start. Comparisons are evaluated with Mann‐Whitney's test.

## Data Availability

The raw data supporting the conclusions of this article will be made available by the authors, when requested.
