# Supplementary material for: Size of HIV‐1 reservoir is associated with telomere shortening and immunosenescence in early‐treated European children with perinatally acquired HIV‐1
Source: J Int AIDS Soc. 2021 Nov 19;24(11):e25847. doi: 10.1002/jia2.25847 (PMC8604380; doi:10.1002/jia2.25847)
Supplement: Supplementary file 1 — Figure S1. Heatmap of the correlations between all the studied clinical, virological and immunological characteristics of the studied cohort. Correlations are colored according to their Spearman's ρ coefficient. § p < 0.1; *p < 0.05; **p < 0.01; ***p < 0.001. Table S1. Comparisons between all the studied clinical, virological and immunological characteristics of the studied population, subgrouped according to the patients' HIV‐1 DNA values or by their age at ART start. Comparisons are evaluated with Mann‐Whitney's test. [file JIA2-24-e25847-s001.docx]

**Figure S1.** Heatmap of the correlations between all the studied clinical, virological and immunological characteristics of the studied cohort. Correlations are colored according to their Spearman’s ρ coefficient. § p<0.1; * p<0.05; ** p<0.01; *** p<0.001.

.
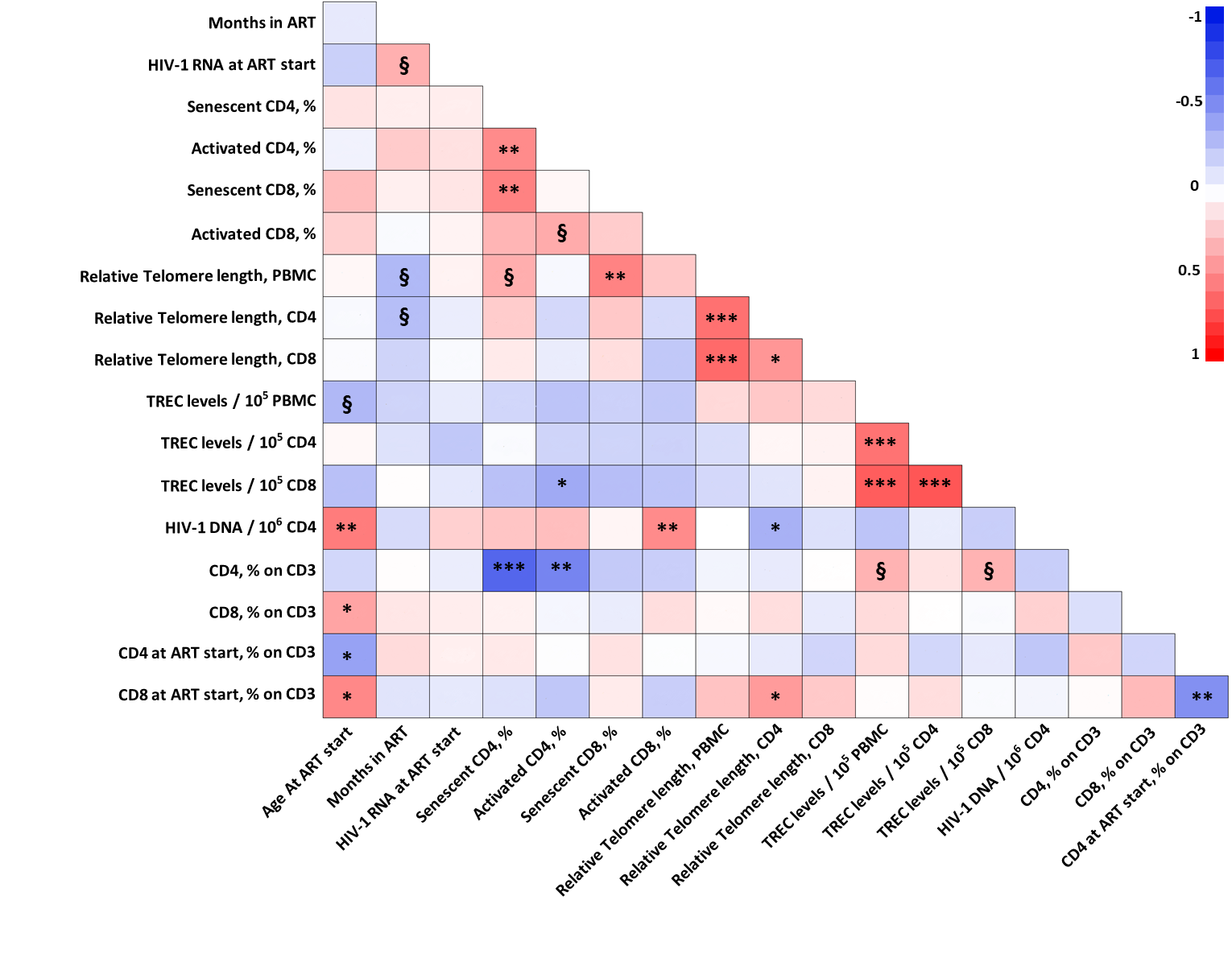


**Table S1.** Comparisons between all the studied clinical, virological and immunological characteristics of the studied population, subgrouped according to the patients’ HIV-1 DNA values or by their age at ART start. Comparisons are evaluated with Mann-Whitney’s test.

|  | **Low/High HIV-DNA subgroups** | | | **Early/Late treatment subgroups** | | |
| --- | --- | --- | --- | --- | --- | --- |
|  | **Low HIV-DNA** | **High HIV-DNA** | **p** | **Early Treated** | **Late treated** | **p** |
| Male/Female | 3/6 | 8/18 | **-** | 8/19 | 4/6 | **-** |
|  |  |  |  |  |  |  |
| **At ART start** |  |  |  |  |  |  |
| Age (months) | 1.46[0.15-4.47] | 5.59[3.65-8.37] | 0.006 | 3.55[0.20-4.57] | 11.02[6.92-19.58] | <0.001 |
| % CD4 | 37[30-51] | 28[18-38] | 0.050 | 36[26-44] | 23[17-37] | 0.092 |
| % CD8 | 36[23-48] | 30[22-40] | 0.536 | 29[24-35] | 39[24-48] | 0.106 |
| Plasma HIV-1 RNA (Log copies/mL) | 4.64[33.81-5.56] | 5.32[4.23-5.73] | 0.257 | 5.04[4.08-5.70] | 5.42[3.94-5.75] | 0.945 |
|  |  |  |  |  |  |  |
| **At sample collection** |  |  |  |  |  |  |
| Age (years) | 13.5[10.7-15.9] | 13.8[8.2-17.1] | 0.756 | 14.0[9.8-16.6] | 9.5[8.6-16.8] | 0.644 |
| Time on ART (years) | 13.4[10.4-15.5] | 13.5[7.4-16,5] | 0.597 | 13.7[9.7-16.4] | 8.6[7.4-15.8] | 0.365 |
| % CD4 | 34.4[29.9-42.7] | 31.1[26.7-38.0] | 0.221 | 35.4[29.8-40.0] | 27.9[24.1-32.9] | 0.045 |
| % CD8 | 13.5[10.5-20.2] | 15.7[13.6-18.8] | 0.391 | 14.5[12.4-18.9] | 17.9[15.4-18.3] | 0.166 |
| HIV-1 DNA copies / 10^6^ CD4 cells | 17.5[0.0-69.3] | 365[184-581] | <0.001 | 89[56-365] | 552[303-1001] | 0.003 |
| Senescent CD4 cells, % | 0.76[0.42-1.80] | 2.15[0.80-3.34] | 0.060 | 1.02[0.45-2.69] | 2.89[1.95-6.31] | 0.047 |
| Senescent CD8 cells, % | 10.0[4.4-14.4] | 14.1[8.6-19.1] | 0.203 | 11.7[7.6-16.5] | 14.2[11.4-26.2] | 0.234 |
| Activated CD4 cells, % | 0.33[0.26-0.38] | 0.46[0.26-0.66] | 0.089 | 0.36[0.26-0.57] | 0.41[0.35-0.50] | 0.955 |
| Activated CD8 cells, % | 1.21[0.84-1.48] | 1.83[1.16-2.35] | 0.030 | 1.38[1.03-1.84] | 1.94[0.99-2.94] | 0.206 |
| TREC levels / 10^5^ PBMC | 2496[1197-4234] | 1199[762-2318] | 0.097 | 1976[1075-3881] | 921[493-1083] | 0.009 |
| TREC levels / 10^5^ CD4 cells | 1762[879-2565] | 1232[855-1739] | 0.416 | 1325[854-2210] | 1228[1019-1580] | 0.873 |
| TREC levels / 10^5^ CD8 cells | 3052[1363-3434] | 1763[833-3313] | 0.281 | 2278[1425-3314] | 1128[486-1671] | 0.042 |
| Relative telomere length in PBMC | 1.32[1.18-1.36] | 1.37[1.18-1.56] | 0.499 | 1.33[1.20-1.40] | 1.35[1.21-1.58] | 0.694 |
| Relative telomere length in CD4 cells | 1.40[1.24-1.62] | 1.32[1.18-1.62] | 0.533 | 1.34[1.21-1.61] | 1.32[1.27-1.37] | 0.729 |
| Relative telomere length in CD8 cells | 1.40[1.27-1.46] | 1.47[1.20-1.58] | 0.597 | 1.40[1.27-1.52] | 1.40[1.16-1.51] | 0.798 |

Values are expressed as median[interquartile range].
